# Successful clinical outcomes following decentralization of tertiary paediatric HIV care to a community-based paediatric antiretroviral treatment network, Chiangrai, Thailand, 2002 to 2008

**DOI:** 10.7448/IAS.15.2.17358

**Published:** 2012-10-11

**Authors:** Rawiwan Hansudewechakul, Thananda Naiwatanakul, Abraham Katana, Worawan Faikratok, Rangsima Lolekha, Vorapathu Thainuea, Michelle S McConnell

**Affiliations:** 1 Chiangrai Prachanukroh Hospital, Ministry of Public Health, Chiangrai, Thailand; 2 GAP Thailand/Asia Regional Program Thailand MOPH – U.S. CDC Collaboration and CDC/Southeast Asia Regional Office, Nonthaburi, Thailand; 3 University of Oxford, Oxford, United Kingdom; 4 Centers for Disease Control and Prevention Atlanta, GA, USA

**Keywords:** Paediatric HIV, HIV care network, paediatric HIV treatment outcomes, task shifting, decentralized HIV care, Thailand

## Abstract

**Introduction:**

Most paediatric antiretroviral treatments (ARTs) in Thailand are limited to tertiary care hospitals. To decentralize paediatric HIV treatment and care, Chiangrai Prachanukroh Hospital (CRH) strengthened a provincial paediatric HIV care network by training community hospital (CH) care teams to receive referrals of children for community follow-up. In this study, we assessed factors associated with death and clinical outcomes of HIV-infected children who received care at CRH and CHs after implementation of a community-based paediatric HIV care network.

**Methods:**

Clinical records were abstracted for all children who initiated ART at CRH. Paired Wilcoxon signed rank tests were used to assess CD4% and virological change among all children. Cox proportional hazard models were used to assess factors associated with death. Treatment outcomes (CD4%, viral load (VL) and weight-for-age Z-score (WAZ)) were compared between CRH and CH children who met the criteria for analysis.

**Results:**

Between February 2002 and April 2008, 423 HIV-infected children initiated ART and 410 included in the cohort analysis. Median follow-up for the cohort was 28 months (interquartile range (IQR)=12 to 42); 169 (41%) children were referred for follow-up at CH. As of 31 March 2008, 42 (10%) children had died. Baseline WAZ (<−2 (*p=*0.001)) and baseline CD4% (<5% (*p=*0.015)) were independently associated with death. At 48 months, 86% of ART-naïve children in follow-up had VL<400 copies/ml. For sub-group analysis, 133 children at CRH and 154 at CHs were included for comparison. Median baseline WAZ was lower in CH children than in CRH children (*p=*0.001); in both groups, WAZ, CD4% and VL improved after ART with no difference in rate of WAZ and CD4% gain (*p=*0.421 and 0.207, respectively).

**Conclusions:**

Children at CHs had more severe immunological suppression and low WAZ at baseline. Community- and tertiary care-based paediatric ART follow-ups result in equally beneficial outcomes with the strengthening of a provincial referral network between tertiary and community care. Nutrition interventions may benefit children in community-based HIV treatment and care.

## Introduction

Approximately 3.4 million children worldwide were living with HIV in 2010, most of them in developing countries [1]. Untreated HIV infection progresses rapidly in children; more than half of those with vertically acquired HIV infection die before their second birthday [2,3]. The use of antiretroviral (ARV) therapy (ART) dramatically reduces HIV-related morbidity and mortality, however, with children often surviving into adulthood in high-income countries [4–7]. Although similar treatment outcomes and survival patterns have been observed in developing countries [8,9], the rollout of ART programs for children has been slow and mortality rates have remained high. As of 2010, 455,700 children worldwide were receiving ART, accounting for only 23% of ART-eligible children, and an estimated 250,000 paediatric AIDS deaths had occurred [1].

High paediatric AIDS death rates have been largely attributed to the difficulties in identifying and treating paediatric HIV in resource-limited settings. Thailand, despite having a national ART program since 2000 [10], has been facing similar challenges. Adult HIV treatment and care has been decentralized to community-based settings, but paediatric ART has generally remained in the tertiary care setting because of a lack of paediatricians and other skilled healthcare providers [11].

**Figure 1:**
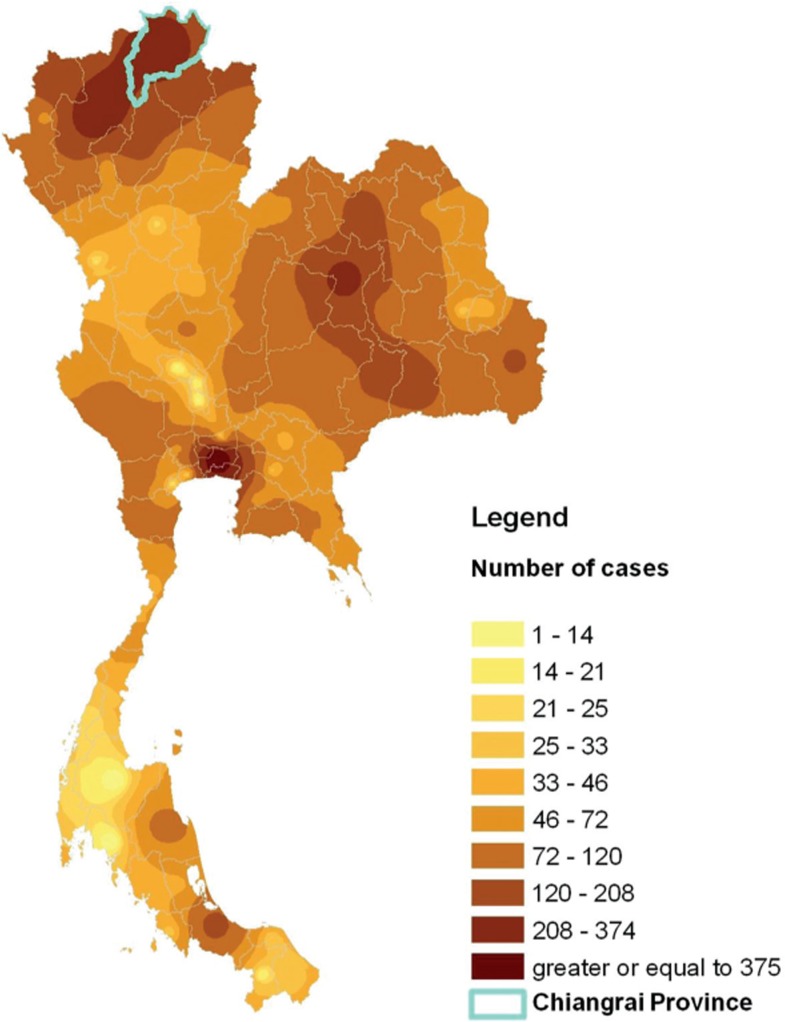
Location and number of paediatric HIV cases on antiretroviral treatment in the Thailand Ministry of Public Health antiretroviral treatment program and Chiangrai province, 2007.

Chiangrai province has one of the highest HIV prevalence rates in Thailand (Figure 1). HIV prevalence rate in women-attending antenatal clinics in Chiangrai peaked at 8.0% in 1995 before declining to 0.9% in 2007; by contrast, national rates were 2.3% and 0.8% in 1995 and 2007, respectively. The mother-to-child transmission rate declined from 24% in 1992 to 19% in 1997 [12,13] and to 10% in 2001 to 2003 following the introduction of zidovudine and then zidovudine plus single-dose nevirapine, for prevention of mother-to-child HIV transmission (PMTCT) [14].

Chiangrai Prachanukroh Hospital (CRH), a tertiary care hospital, is the referral hospital for 16 community hospitals (CHs) in the province. With increased access to ART, the paediatric HIV caseload at CRH increased from fewer than ten patients in 2002 to more than 300 children in 2004. Each year, 70 to 100 additional children become eligible for ART [15]. This increase has a significant impact on staff workload, and the centralization of services posed challenges to families in rural areas, including high costs for transportation and absences from school and work. To address these needs, CRH strengthened a provincial paediatric HIV care network by training CH staff to receive referrals of children for community follow-up. This study assessed factors associated with death and the clinical outcomes of children who received care at CRH and CHs after implementation of a community-based paediatric HIV care network.

## Methods

### Community-based paediatric HIV care and treatment network

Between 2004 and 2006, CRH trained and mentored its 16 referral network CHs to provide holistic paediatric ART. CH teams consisted of at least two healthcare professionals, including nurses/counsellors, people living with HIV (PLHIV), pharmacists, and physicians. CRH trainings supplemented routine national trainings and included a review of paediatric HIV treatment and care; observation of service provision at CRH; demonstration of clinical and adherence monitoring; and use of case record forms and education materials. CRH and CH teams provide the same standard paediatric HIV treatment and care following national guidelines [16], including clinical assessment and laboratory monitoring, screening for opportunistic infections (OIs), prophylaxis, adherence assessments, and routine ARV dose adjustment and psychosocial support. In complicated cases, such as severe adverse drug reactions or ARV resistance, CHs consult by telephone with the CRH team or refer children back to CRH for management. Due to the limited number of physicians in most CHs, nurses usually provide leadership and clinical care, often prescribing ARVs under general physician attendance. Pharmacists and trained PLHIV promote ARV adherence, monitor adverse drug reactions and provide psychosocial support. After referring children for CH follow-up, mentoring and regular communication between CRH and CH occur through teleconferences, individual case consultations, email, medical record review, PLHIV home visit record review and on-site visits. Typically, a CRH team visited each CH three to six months after training to ensure that care and treatment was consistent with guidelines.

All children in this analysis initiated ART following the Thai national ART guideline at CRH. Children residing in community districts were referred for follow-up at CHs when they were clinically stable, as evidenced by the absence of OIs and improvements in weight and CD4%. Additional criteria for referral included caregiver willingness to have the child receive ART at the CH. Children followed up at CHs were scheduled to be seen by a paediatrician at CRH every six months for clinical and CH service quality monitoring (or sooner, if they developed complications or signs of treatment failure).

### Assessment of treatment outcomes

Clinical records were abstracted for all children initiated ART at CRH and followed up at either CRH or a CH between February 2002 and March 2008. Children without follow-up data were excluded.

Adherence, weight and CD4% were assessed every six months. Optimal adherence was defined as at least 95% of pills or liquid medications taken (based on pill counts or volume measurement). Children who did not bring back or lost their pills were treated as missing data. The US Centers for Disease Control and Prevention (CDC) classification of HIV-related disease at ART initiation was used [17]. Weight-for-age Z-scores (WAZ) were calculated using Thailand national standards for height and weight. Three WAZ categories were created based on World Health Organization (WHO) references: “normal weight” (>−2), “moderately underweight” (−3 to −2) and “severely underweight” (<−3). Changes in WAZ, CD4% and viral load (VL) were calculated over the 48-month follow-up period. As routine VL testing was not initially available, baseline VL was available in only a subset of children who were participants in a separate prospective observational cohort study. The median baseline VL was determined for children who had their first VL test within one month of enrolment at CRH, and the proportion of children with VL<400 copies/ml was determined at 12 month intervals for 48 months. Virological response was compared in children who had a baseline and at least one follow-up VL result. VL results were included if they fell within a six-month window of the 12-month interval follow-up time point. Overall crude mortality rates were calculated, as well as early (death occurred within the first 90 days), intermediate (death between 91 and 180 days) and late (death after 180 days) mortality rates.

**Figure 2:**
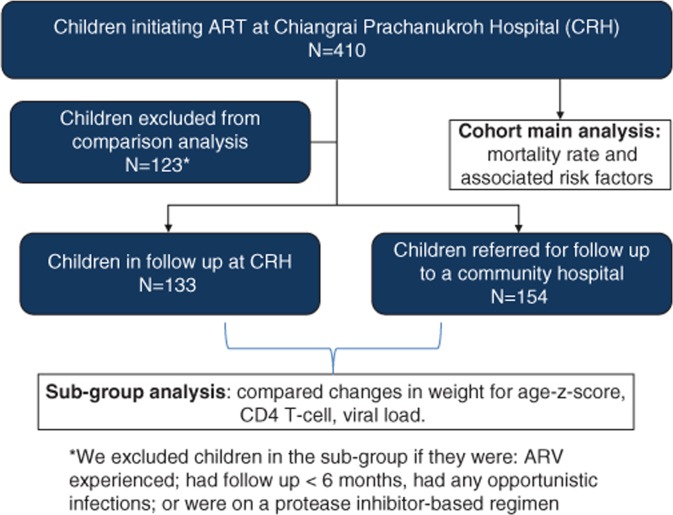
HIV-infected children participating in the HIV care network, Chiangrai, Thailand, 2002 to 2007.

Sub-group analysis was performed to compare treatment outcomes (WAZ, CD4% and viral suppression (VL<400 copies/ml)) from children followed up at CRH and CHs. To reduce potential bias of difference in virological response, children were excluded from this sub-analysis (*n=*123) if they had a history of previous ART (*n=*42), initiated protease inhibitor (PI)-based ART (*n=*16) or were on ART for less than six months (*n=*65) (Figure 2). Mortality was not compared because of the absence of any deaths in the CH group.

**Figure 3:**
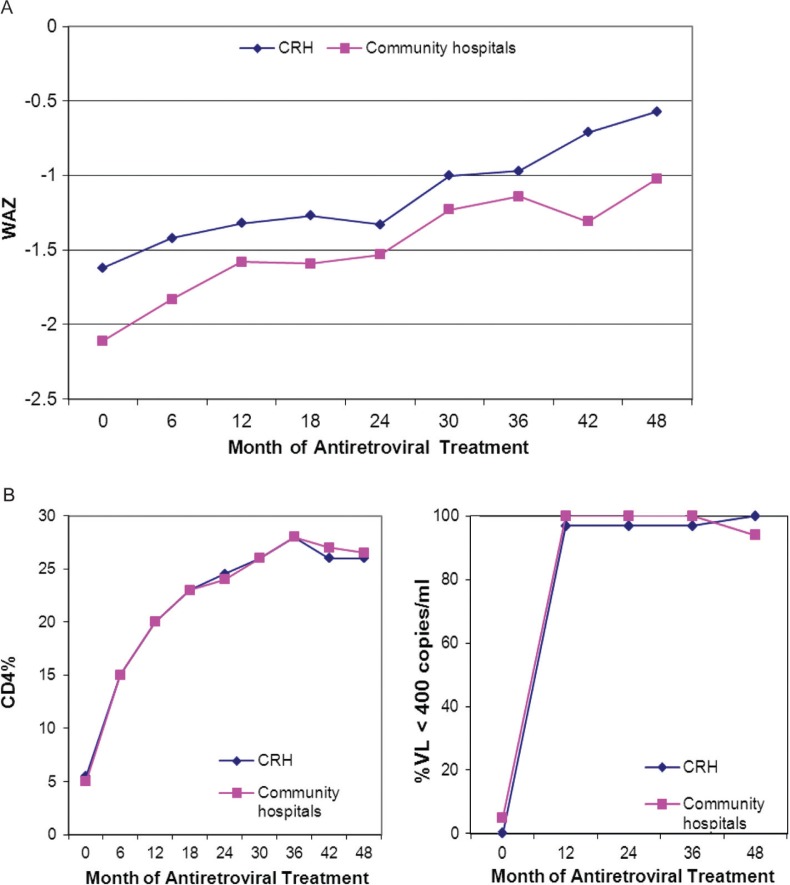
(a) Weight-for-age change in response to ART among children in follow-up at Chiangrai Prachanukroh Hospital (CRH) and community hospitals, Chiangrai, Thailand, 2002 to 2007. (b) CD4 and virological responses to ART among children in follow-up at Chiangrai Prachanukroh Hospital (CRH) and community hospitals, Chiangrai, Thailand, 2002 to 2007. Note: Weight for age Z-score (WAZ) was significantly different (*p*<0.05) between patient groups at CRH and community hospitals at months 0 to 24.

### Statistical analysis

Data was extracted from a clinic-based Microsoft Access database and analyzed using STATA (version 10, Stata Corporation, College Station, TX) and SPSS (version 15, SPSS Inc., Chicago, IL). Categorical variables were compared using the Pearson χ^2^ test, and continuous variables were compared using *t*-tests and non-parametric Wilcoxon rank-sum tests as appropriate. Interquartile ranges (IQRs) were calculated for median values, and 95% confidence intervals (CIs) were calculated for means. Two-sided *p*-values were analyzed, with *p*≤0.05 considered significant. Multiple imputations were done for CD4, clinical staging and WAZ to account for missing data. Imputation results were not significantly different; therefore, only complete case results are presented.

Child-years of follow-up were calculated from the date of ART initiation at CRH until the earliest of the following dates: (a) death, (b) lost to follow-up or (c) 31 March 2008 (the day when data was censored). Referral time was calculated from the date of ART initiation to the date of referral to CHs. Survival rates at 12-month intervals were calculated using the Kaplan Meier estimator. Cox proportional hazard regression models with backward stepwise regression were fit to identify risk factors for mortality. Schoenfeld residuals and scaled-Schoenfeld residuals were plotted for both covariate-specific and global tests to assess validity of model assumptions. There was no evidence of violation of model assumptions in all reported analyses. Clinical staging and baseline CD4 were collinear; therefore, only CD4 was included in the model. Spearman correlations were used to assess the association between duration of treatment at CRH and WAZ and CD4% outcomes in CH sub-groups.

This study was approved by the CRH Institutional Review Board. CDC human subjects’ office approved this project as research not involving human subjects. All identifiers were removed from the dataset prior to analysis.

## Results

### Entire study cohort

During the study period, 423 children initiated ART at CRH. Three hundred and thirty-four (86%) were born before 2000, when the national PMTCT program was implemented, and 382 (92%) initiated ART in or after 2003, when ART became accessible nationally. Thirteen (3%) children without follow-up data were excluded from the analysis. Excluded children had similar age, baseline CD4% and clinical staging and were new to ART initiation. The baseline characteristics of 410 children are shown in Table 1. Two hundred and thirty-one (56%) and 70 (17%) children had follow-up data for more than 24 and 48 months, respectively. As of 31 March 2008, 42 children (10%) had died and 368 (90%) remained on ART. There was no lost to follow-up documented during the study period. One child refused to continue ART but remained in follow-up; eight children were transferred to other provincial clinics when they changed residence.

**Table 1:** Baseline^a^ characteristics of children initiating ART at Chiangrai Prachanukroh Hospital (CRH) and among those referred for follow-up^b^ at CRH or community hospitals, Chiangrai, Thailand, 2002 to 2007

Variable	Entire cohort initiating ART (*n*=410)^c^	CRH follow-up (*n*=133)^c^	Community hospital follow-up (*n*=154)^c^	*p*^d^
Gender, *n* (%)				
– Male	177 (43)	61 (46)	66 (43)	0.610
Baseline age, years				
– Median (IQR)	8.6 (6.5 to 10.6)	8.0 (6.7 to 11.3)	8.0 (6.8 to 10.3)	0.455
Baseline HIV stage (%)				
– N or A	131 (32)	51 (38)	50 (32)	
– B	73 (18)	30 (23)	28 (18)	
– C	125 (31)	33 (25)	61 (40)	0.05
Baseline WAZ score (%)				
– <−3 SD	67 (16)	19 (14)	28 (18)	
– −3 to −2 SD	110 (27)	30 (23)	52 (34)	
– >−2 SD	226 (55)	84 (63)	74 (48)	0.034
– Median (IQR)	−1.9 (−2.7 to −1.1)	−1.6 (−2.6 to −0.8)	−2.1(−2.8 to −1.5)	0.001
Baseline CD4% (%)				
– <5%	158 (39)	50 (38)	67 (44)	
– >5%	224 (55)	76 (57)	79 (51)	0.302
– Median (IQR)	6 (2 to 13)	6 (2 to 12)	5 (2 to 11)	0.303
Baseline viral load (copies/ml)				
Median (*n*)	100,000 (*n*=79)	133,570 (*n*=38)	80,400 (*n*=22)	0.167
(IQR)	(18,629,234,420)	(49,380, 230,700)	(17,622, 153,022)	
CD4% (at referral^e^)				
– Median (IQR)	—	20 (14 to 24)	20 (16 to 26)	0.342
WAZ score (referral^e^)	—			
– Median (IQR)		−1.3 (−2.0 to −0.7)	−1.6 (−2.2 to −1.2)	0.003
Follow-up time, months				
– Median (IQR)	28 (12 to 42)	35 (16 to 48)	29 (20 to 40)	0.417

a Includes baseline characteristics for all variables with the addition of CD4% and weight-for-age Z score (WAZ) at time of referral.

b Follow-up groups include children eligible for CH follow-up and a comparable group who stayed in follow-up at CRH and meet inclusion criteria (as defined in methods).

c Missing data; therefore, numbers and percents may not add to total.

d *p*-value for Pearson χ^2^ test or Wilcoxon rank-sum test as appropriate to compare CRH and community group.

e 12-month time point assessment taken based on the median referral time of 14 months after ART initiation.

One hundred and sixty-eight (41%) children were referred to CHs for follow-up, contributing to a median follow-up time of 18 months (IQR 12 to 30 months) in CHs. Of these, only six (3.5%) were referred back for follow-up at CRH: three because of treatment failure and three for management of adverse events. After the CH referral program started in October 2004, the median referral time decreased from 31 months (IQR 20 to 39) in children initiating ART before October 2004 to 13 months (IQR 8 to 81) for children initiating ART between October 2004 and September 2006 and to eight months (IQR 3 to 11) for children initiating ART after September 2006.

When they initiated ART at CRH, 366 (90%) children were ART-naïve. The median age at ART initiation was 8.6 years (IQR 6.5 to 10.7 years), with only 12 (2.9%) children younger than two years. Most children had advanced disease at ART initiation as indicated by their weight, clinical stage and immunological status (Table 1). Three hundred and forty-one (83%) initiated a nevirapine-based regimen, 53 (13%) an efavirenz-based regimen and 16 (4%) a PI-based regimen; all those on a PI-based regimen were research study participants who transferred to the CRH program or were born to single-dose nevirapine-exposed mothers. Three hundred and four (74%) children had at least one parent died, and 149 (36%) had both parents died.

### Treatment outcomes

The increase in WAZ score of all children in the cohort was consistent, improving from a baseline median IQR of −1.9 (−2.7 to −1.1) to −1.4 (−1.9 to −0.5) and −0.8 (−1.6 to −0.3) at 24 and 48 months. The proportion of children who were moderately and severely underweight decreased from 27% and 17% to 14% and 1.8%, respectively, at 24 and 48 months. Adherence data was available in 89 to 97% of children in follow-up. Of those, 96 to 100% reported at least 95% adherence throughout the follow-up period. The median CD4 lymphocyte percent improved from a baseline of 6% (IQR 2 to 13%) to 24% (IQR 20 to 29%) at 24 months and 26% (IQR 22 to 31%) at 48 months. Greatest improvement was in the first six months of treatment, during whichCD4% more than doubled to a median of 15% (IQR 10 to 22%). Overall, the proportion of children with severe immune suppression (CD4<15%) decreased from 80% at ART initiation to 7.1% at 24 months and 4.8% at 48 months. However, less than half (46%) of the children who enrolled with CD4 of<5% had CD4%≥25% at 48 months. The virological response among ART-naïve children on ART in follow-up at 12, 24, 36 and 48 months with VL<400 copies/ml was 158/174 (91%), 173/186 (93%), 143/154 (93%) and 121/140 (86%), respectively (Figure 3b).

Forty-two children (10%) died over the 1,016 child-years of follow-up, representing a crude mortality rate of 4.1/100 child-years of follow-up (95%CI, 3.0 to 5.6). Most deaths occurred soon after ART initiation: 25 (60%) children within the first 90 days. Seventeen (40%) children died after 90 days, of which 7 (41%) died after 180 days. The early mortality rate was 24.6/100 child-years (95% CI 15.8 to 36.7), the intermediate mortality rate was 2.1/100 child-years (95% CI 1.2 to 3.3) and the late mortality rate was 0.9/100 child-years (95% CI 0.4 to 2.2).

The cause of death was recorded for 30 (71%) of the 42 children who died. Fourteen deaths were due to bacterial or viral respiratory tract infections, four to tuberculosis or suspected tuberculosis, two to penicillosis, two to cryptococcus, one to cytomegalovirus, one to sepsis, three to other HIV-related causes and three to non-HIV-related causes. None of the children died while in follow-up at CHs or after being referred back to CRH from a CH.

In univariate analysis of factors associated with mortality, orphan status and baseline WAZ score, clinical stage and immunologic status were significantly associated with death. In multivariate analysis, only severe malnutrition (WAZ<−3 SD) and severe immune suppression (CD4<5%) at baseline remained independent predictors of mortality (Table 2). Orphan status and clinical stage were not assessed in the multivariate analysis because of non-significance in tests for linear trend (*p=*0.37) and collinearity with immune category, respectively.

**Table 2:** Factors associated with mortality among all children initiating ART at Chiangrai Prachanukroh Hospital, Chiangrai, Thailand, 2002 to 2007

		Unadjusted analysis		Adjusted analysis	
			
Baseline variable	Number of deaths (%)	HR (95%CI)	*p*	HR (95%CI)	*p*
Sex					
Male	21 (11.9)	1		1	
Female	21 (9.0)	0.7 (0.4 to 1.3)	0.311	0.7 (0.3 to 1.5)	0.342
Age (years)					
<5	6 (9.4)	1		1	0.390
5–9.9	20 (9.8)	1.1 (0.4 to 2.7)	0.880	1.9 (0.4 to 8.7)	0.477
≥10	16 (11.3)	1.2 (0.6 to 2.3)	0.585	1.4 (0.6 to 3.6)	
Orphan status					
Both parents alive	16 (15.1)	1		Not included^a^	
One parent died	9 (5.8)	0.4 (0.15 to 0.79)	0.014		
Both parent died	17 (11.4)	1.2 (0.64 to 2.24)	0.579		
ARV regimen					
NVP-based	38 (11.1)	1			
EFV-based	3 (5.7)	0.5 (0.14 to 1.52)	0.205		
PI-based	1 (6.3)	0.8 (0.1 to 6.3)	0.838		
Median WAZ score					
>−2 SD	11 (4.9)	1		1	0.470
−2 to −3 SD	16 (8.2)	1.7 (0.7 to 4.2)	0.224	1.5 (0.5 to 4.0)	0.000
<−3 SD	11 (23.9)	4.3 (2.2 to 8.2)	0.000	3.4 (1.3 to 9.1)	
CD4 status					
>5%	8 (3.6)	1		1	
<5%	28 (17.8)	5.0 (2.4 to 10.0)	0.000	3.1 (1.2 to 7.7)	0.015
CDC clinical stage					
Asymptomatic/mild	2 (15)	1			
Moderate	4 (5.5)	3.6 (0.8 to 19.5)	0.142	Not included^b^	
Severe	21 (16.8)	6.6 (2.5 to 17.2)	0.000		

Note: Cox proportional hazards models between baseline variables and death.

a Not included due to non-significance in test for linear trend.

b Not included in the multivariate analysis due to co-linearity with CD4 status.

### Sub-groups analysis

In the sub-groups identified for comparison, the 133 and 154 children who received follow-up at CRH and CHs, respectively, had similar ages, sex, duration of follow-up, median baseline CD4%, median baseline VL and orphan status (Table 1). CH children initiated ART with a significantly lower median WAZ score than CRH children (*p=*0.001); clinical stage had borderline significance (*p=*0.05) (Table 1). At referral, median time on ART was 14 months (IQR 3 to 55). There was no correlation between duration of ART at CRH prior to referral and changes in WAZ and CD4% at 12, 24, 36 and 48 months.

Adherence data was available for 73 to 93% of CRH children and 83 to 95% of CH children. CH and CRH children had similar adherence levels (95 to 100% and 93 to 100% of children had ≥95% adherence, respectively, over the 48 months).

Thirty-eight (29%) CRH children and 22 (14%) CH children had VL data at baseline and at least one follow-up visit. At 12, 24 and 36 months follow-up, only 1 each out of the 37, 35 and 35 CRH children and none of the 20, 16 and 18 CH children, respectively, had VL>400 copies/ml. At 48 months, none of the 27 CRH children and 1 of the 18 CH children had VL>400 copies/ml [Figure 3b]. There was no difference in the rate of weight gain between CH and CRH children (mean difference in WAZ score gain was 0.88 and 1.06 for CRH and CH children, respectively, p=0.421). However, CH children not only had lower WAZ at baseline, but also continued to have lower WAZ through 48 months, although the difference was only significant through 24 months (p=0.042) [Figure 3]. There was no difference in CD4% gain between CRH and CH children (median difference was 19% and 21% [p=0.207], respectively) [Figure 3].

## Discussion

This study describes the implementation and treatment outcomes of a paediatric HIV care network linking public sector provincial and community ART programs in northern Thailand. Findings suggest that despite difficulties associated with the administration of ART for HIV-infected children, it is possible to achieve and sustain beneficial outcomes in a decentralized ART program in resource-limited settings by strengthening a network of tertiary- and community-based care providers. This is demonstrated by high retention rates and clinical and immunological improvements, despite the fact that most children initiated ART only at the advanced disease stage.

The study cohort represents a first group of children with perinatally acquired HIV-infection in Thailand before the national PMTCT was implemented in 2000 (86% born before 2000). The older age and severe immunological suppression at ART initiation in this cohort were likely due to late diagnosis and referral for ART initiation; the national program for early infant diagnosis started in 2007, and full access to the national ART program started in 2003 to 2004 [10]. Therefore, 92% of children in this cohort initiated ART in or after 2003. The finding that children at CHs had significantly lower baseline WAZ and more advanced stage HIV infection is likely due to the abovementioned reasons and the lower in socio-economic status of families living in more rural areas.

In this study, adherence level remained high among children in follow-up at both CRH and CHs, and much of the success of the paediatric care network may be attributed to good adherence. Adherence requires special attention to children because of the need for caregiver support, paediatric formulations and palatable medications with easy dosing schedules. Paediatric treatment is made more challenging as a result of the high level of adherence required to prevent sub-optimal viral suppression, a risk factor for development of HIV drug resistance [18,19] and mortality [20]. Although comparison across studies should be done with caution, this study reported better adherence than most paediatric treatment programs, with several studies reporting 50 to 80% adherence [21–23]. The intensity and support given to patients and caregivers in this program may explain the high level of adherence and suggest that multiple approaches combining educational and behavioural components are useful in achieving desirable adherence levels [24].

Weight gain, VL and CD4% responses to ART in this cohort compare favourably to other studies. A prospective cohort study of 107 children in the neighbouring Chiang Mai province reported similar responses [25]. In the Paediatric AIDS Clinical Trials Group (PACTG) 219 study, 33% of children who initiated treatment with CD4<5% reached normal levels after three years of treatment, compared to 45% after four years in our study [26]. Both the Chiang Mai and PACTG studies were conducted in settings where clinical care was provided by paediatricians; obtaining similar results in a community-based program with non-paediatric and non-physician staff is quite encouraging.

The mortality rate of 4.1/100 child-years of follow-up with most deaths occurring within the first three months of treatment was comparable to other paediatric cohorts in resource-limited countries [8,9,27,28–30], as was the association between advanced HIV-related disease and mortality [9,20,23,24]. These findings underscore the importance of early identification of HIV-infected children and timely initiation of ART. However, the finding that most children who stayed in follow-up had viral suppression and remained on first-line ART through 48 months confirms the reported high level of ART adherence and the importance of intensive adherence support in this program [25]. The absence of deaths among children followed up at CHs is not surprising given that patients were referred when clinically stable, typically at least six months after ART initiation.

Although not a direct comparison of tertiary and CH treatment outcomes, study results are of interest, especially given the strategy of WHO [31] to shift tasks to less specialized healthcare workers and thereby increase access to care. The success of this model highlights its potential for other settings with high HIV burdens and limited resources, such a sub-Saharan Africa. Similar findings on improvement of clinical and virological outcomes for decentralized care for both adults and children have been reported in several African countries [32–35]. Of note, the provision of adherence support by non-clinical staff, mainly PLHIV, could off-set the limited number of trained healthcare workers. The level of adherence support in this program, however, is intensive, and replicating it in less developed countries with higher HIV burdens may prove challenging.

Although these results compare favourably with other published literature, there were several limitations. Missing data was probably due to a retrospective study designed and utilized routine program data. However, in the multiple imputative analyses, results and overall interpretations were not changed. Some data, such as VL, were limited because of programmatic reasons, and findings may not be generalizable to the entire cohort. Although the rates and causes of mortality are consistent with other studies, the incompleteness of data limits the conclusions about cause-specific mortality. Additionally, although efforts were made to objectively compare treatment outcomes between children in follow-up at CRH and CHs, the unmatched design, differences in baseline WAZ, referral of patients based on the clinician's judgment and the willingness of the caregiver to have the child receive ART in the CH, all may have limited the ability to compare the two groups. Nevertheless, baseline age, gender and clinical characteristics, with the exception of WAZ, were similar between the groups. Finally, few young children and infants in the entire cohort and only ART-naïve children were included in the sub-group analysis; thus, findings may not be generalizable to younger and ART-experienced children. The median age of this cohort and the absence of young infants reflect the increased uptake of PMTCT interventions [36] with only 41 children (10%) in this cohort born with HIV-infection after 2000.

In summary, this study demonstrates improvements in clinical immunological and virological outcomes in children followed up in a tertiary and community-based ART network, over a long follow-up period. This study also suggests that paediatric HIV care can be shifted from tertiary to community-based settings when tertiary care staff initiate ART, train community teams and consistently monitor and support community staff work. This may help increase patient's access to treatment, while at the same time reducing workload at overburdened tertiary hospitals.
